# Inadvertent subcutaneous injection of COVID-19 vaccine

**DOI:** 10.1136/postgradmedj-2021-139870

**Published:** 2021-02-15

**Authors:** Jia Yu Ng

**Affiliations:** Sunderland Eye Infirmary, Sunderland SR2 9HP, UK

COVID-19 has been the single greatest public health emergency in the history. The global demand for vaccine vastly outstrip available supply during this scale-up period. There is therefore a need to train up more vaccinators to maximise vaccine uptake in short time period.

Like most other vaccines, the COVID-19 vaccine should be given intramuscularly. Muscles have good vascularity, and therefore allowing injected drug to reach systemic circulation quickly, bypassing the first-pass metabolism.^1^ Intramuscular injection of the deltoid muscle should be given along a line drawn vertically downwards from the mid acromion.^2^ The manufacturers advise that the vaccine should not be injected intravascularly, subcutaneously or intradermally.^3^ Injecting a vaccine into the layer of subcutaneous fat with poor vascularity resulting in slow mobilisation and processing of antigen leading to vaccine failure.^4^ The antigen may take longer to reach the circulation after being deposited in fat, delaying presentation to T and B cells that are essential for immune response. In addition, there is a risk that the antigens may be denatured by enzymes if they remain subcutaneously for prolonged period. Subcutaneous injections can lead to localised cellulitis, granuloma formation and abscess.

The COVID-19 vaccine has shown to have high efficacy if given correctly intramuscularly. Subcutaneous injection can happen inadvertently (figure 1), affecting efficacy of vaccination and potentiate local adverse events. It is vital importance to reinforce intramuscular injection training with competency assessment at intervals in order to maximise efficacy and maintain public confidence.

**Figure 1:**
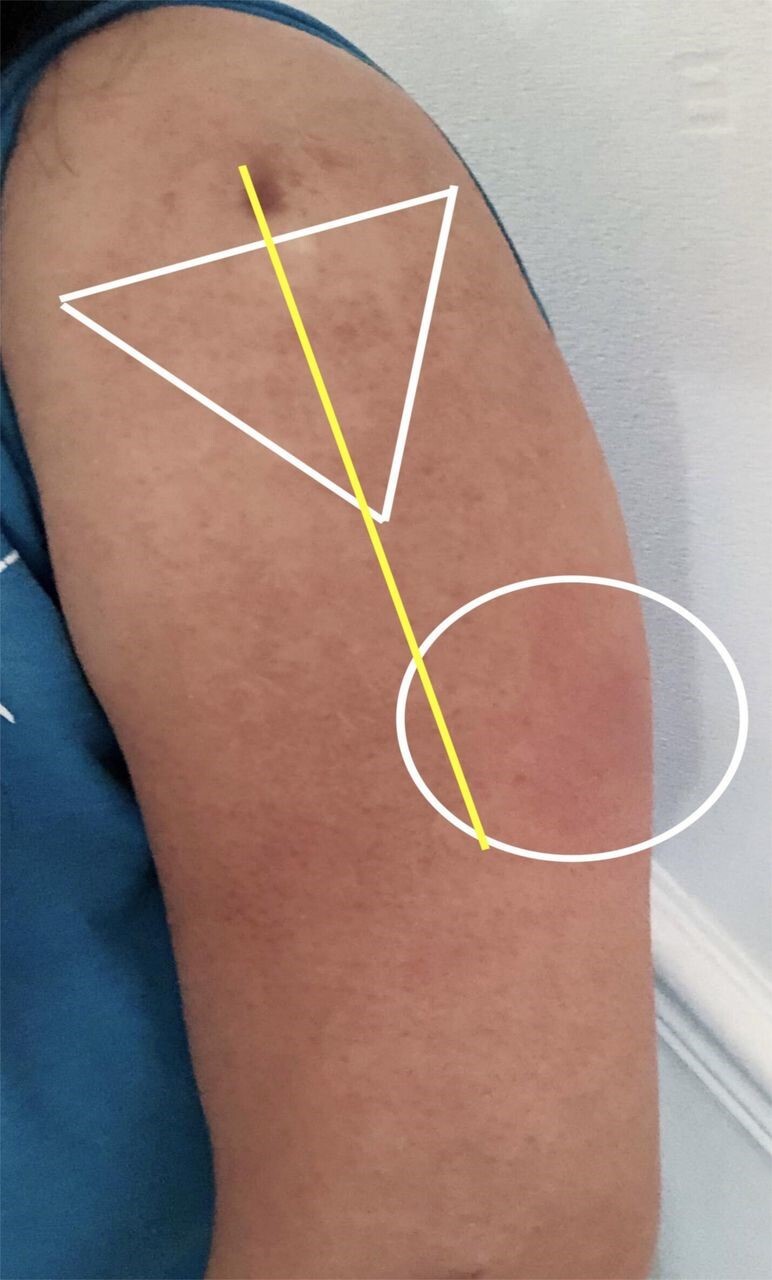
Large localised injection site reaction on posterior aspect of the upper arm following inadvertent subcutaneous injection of COVID 19 vaccine (white circle). Intramuscular injection to deltoid should be along perpendicular line from the mid-acromion (yellow line) and within safe triangle.

## References

[1] Zuckerman JN . The importance of injecting vaccines into muscle. Different patients need different needle sizes. BMJ 2000;321:1237–8. 11082069 10.1136/bmj.321.7271.1237 PMC1118997

[2] Cook IF . Best vaccination practice and medically attended injection site events following deltoid intramuscular injection. Hum Vaccin Immunother 2015;11:1184–91. 25868476 10.1080/21645515.2015.1017694 PMC4514326

[3] GOV.UK . Information for Healthcare Professionals on Pfizer/BioNTech COVID-19 vaccine [Internet], 2021. Available: https://www.gov.uk/government/publications/regulatory-approval-of-pfizer-biontech-vaccine-for-covid-19/information-for-healthcare-professionals-on-pfizerbiontech-covid-19-vaccine

[4] Cook IF . Subcutaneous vaccine administration – an outmoded practice. Hum Vaccin Immunother 2020;97. 10.1080/21645515.2020.1814094 PMC8086591 32991241

